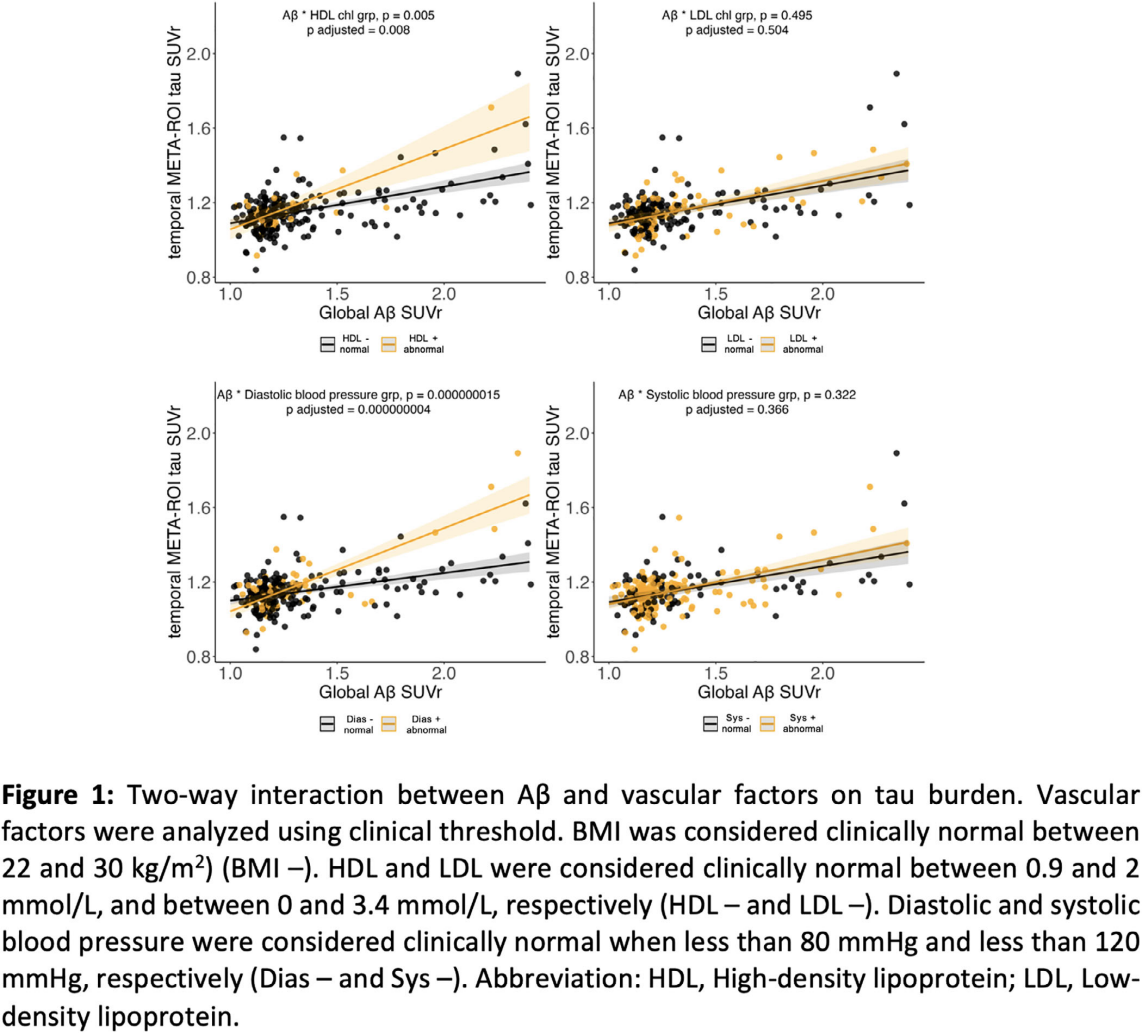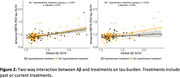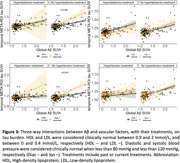# Vascular risk factors modulate the association between amyloid and tau PET in cognitively normal patients

**DOI:** 10.1002/alz70856_098216

**Published:** 2025-12-24

**Authors:** Valentin Ourry, Ting Qiu, Daniel C Bowie, John C.S. Breitner, Judes Poirier, Sylvia Villeneuve, For the PREVENT‐AD Research group

**Affiliations:** ^1^ Douglas Mental Health University Institute, Centre for Studies on the Prevention of Alzheimer's Disease (StoP‐AD), Montréal, QC, Canada; ^2^ Integrated Program in Neurosciences, McGill University, Montréal, QC, Canada; ^3^ McGill University, Montreal, QC, Canada; ^4^ StoP‐AD Centre, Douglas Mental Health Institute Research Centre, Montreal, QC, Canada; ^5^ Douglas Mental Health University Institute, Montreal, QC, Canada

## Abstract

**Background:**

Vascular risk factors have been associated with increased risk of Alzheimer's disease (AD) dementia. Previous studies showed mixed and complex interactions between vascular pathology and AD. In a longitudinal study of cognitively normal participants, we evaluated whether individual vascular risk factors are associated with amyloid and/or tau burden. We also investigated whether these factors and their treatments influence the association between amyloid and tau pathology.

**Methods:**

We performed [18F]‐NAV4694 and [18F]‐AV1451 positron emission tomography on 241 older adults (age 68.3 ± 5.1 years, 69.3% female) from the PREVENT‐AD cohort. All participants had been cognitively unimpaired at baseline. Longitudinal scans were available for 115 persons (4.4 ± 0.6 years follow‐up). We examined the association between individual vascular factors (ApoE status, BMI, cholesterol, blood pressure) and global Aβ and/or temporal META‐ROI tau burden, as well as their annual change. We then examined two‐way interactions between global Aβ and these vascular factors (using clinical‐categorical measures) or treatments as predictors of tau burden. Finally, we explored three‐way interactions that included both vascular factors and medical treatments. All analyses were adjusted for age, sex, and education.

**Results:**

Among the individual vascular risk factors, only ApoE4 status was significantly associated with amyloid burden (*p* < 0.001) and its annual change (*p* = 0.002). ApoE4 status also predicted tau burden (*p* < 0.001), but its association with annual change in tau was at a trend level (*p* = 0.088; not shown). At abnormal levels of HDL cholesterol, and diastolic blood pressure, there was a stronger increase in temporal Meta‐ROI tau‐PET at any given level of amyloid PET (Figure 1). Furthermore, stronger amyloid‐related increase in tau was observed in patients untreated for hypertension but not treated patients (Figure 2). Three‐way interactions showed that HDL cholesterol and diastolic blood pressure were modulatory factors only in the hypertension untreated patients, suggesting that hypertension treatment alleviates the influence of vascular risk factors on tau pathology (Figure 3).

**Conclusions:**

Hyperlipidemia and hypertension, if untreated, are associated with accelerated amyloid related increase in tau pathology in AD.